# Case series of Type 1 Brugada pattern provoked by exercise: a role for diagnostic treadmill stress testing

**DOI:** 10.1093/ehjcr/ytaf640

**Published:** 2025-12-12

**Authors:** Ojas H Mehta, Angela Ambrosio, Lindsay Burnell, Laura Arbour, Michael J Thibert, Andrew D Krahn, Richard Leather, Martin van Zyl

**Affiliations:** Department of Cardiology, Royal Jubilee Hospital, Unit 300-3680 Uptown Blvd, Victoria, BC V8Z 0B9, Canada; School of Medicine, University of Galway, University Road, Galway H91 TK33, Ireland; Division of Medical Genetics, Department of Pathology, Laboratory Medicine, and Medical Genetics, Island Health, Victoria, BC V8Z 6R5, Canada; Department of Medical Genetics, University of British Columbia, Life Sciences Centre, 2350 Health Sciences Mall Room, Vancouver, BC V63 1Z3, Canada; Department of Cardiology, Royal Jubilee Hospital, Unit 300-3680 Uptown Blvd, Victoria, BC V8Z 0B9, Canada; Heart Rhythm Services, Department of Medicine, St-Paul’s Hospital, University of British Columbia, Vancouver, BC V5Z 1M9, Canada; Department of Cardiology, Royal Jubilee Hospital, Unit 300-3680 Uptown Blvd, Victoria, BC V8Z 0B9, Canada; Department of Cardiology, Royal Jubilee Hospital, Unit 300-3680 Uptown Blvd, Victoria, BC V8Z 0B9, Canada

**Keywords:** Brugada pattern, Stress test, Exercise treadmill test, Case series

## Abstract

**Background:**

Brugada syndrome (BrS) is a rare cardiovascular condition that can lead to life-threatening arrhythmias. The *SCN5A* gene, most implicated in heritable BrS, is found in only 20% of those who have the clinical phenotype seen with BrS. Establishing the diagnosis of BrS can be challenging as the electrocardiogram (ECG) pattern that is diagnostic for the syndrome is often transient. Typically, a pharmacological challenge using a Class 1 antiarrhythmic is used to provoke a Type 1 Brugada pattern on ECG that may otherwise be concealed.

**Case summary:**

We describe a case series of three unrelated patients who underwent an exercise treadmill test (ETT) according to standard Bruce protocol. Each patient demonstrated a Type 1 Brugada pattern most apparent in the early recovery phase immediately after peak exercise but not at baseline.

**Discussion:**

This case series highlights the value of ETT as a diagnostic aid for BrS. We suggest that ETT could be considered as a routine means of provoking a Brugada ECG pattern.

Learning points

- Diagnostic ECG patterns in BrS are often transient and may need to be provoked.
- Exercise treadmill testing is not currently performed as a diagnostic workup for BrS. From the presented case studies, we suggest treadmill testing could be considered as a means of provoking a Brugada ECG pattern in order to aid diagnosis.
- Healthcare providers should be vigilant when interpreting treadmill testing, particularly in patients who have suspicion of BrS or are on sodium channel blockers that could potentially provoke a Brugada pattern on ECG.

## Introduction

Brugada syndrome (BrS) is a rare heart rhythm condition with an estimated prevalence of 1 in 5000 to 1 in 2000.^1^ This syndrome is characterized by a distinct electrocardiogram (ECG) pattern consisting of an atypical right bundle branch block and right precordial cove-shaped ST elevation. Some of the consequences of this syndrome include syncope or sudden cardiac death from potential ventricular arrhythmias. Of patients with structurally normal hearts, BrS may be responsible for an estimated 20% of sudden arrhythmic deaths.^2^ The ECG pattern may occur spontaneously, yet most patients display a concealed form of this syndrome.^3^

The diagnostic hallmark of BrS is a specific ECG pattern. Three subtypes have been described: Type 1 encompasses a cove-shaped ST elevation in right precordial leads, with J wave or ST elevation of 2 mm (mV) at its peak, followed by a negative T wave; Type 2 has a J wave amplitude of 2 mV, which leads to a gradually descending ST elevation remaining 1 mV above the baseline, followed by a positive or biphasic T wave that results in a saddleback configuration; and Type 3 is defined by a saddleback or coved right precordial ST elevation < 1 mm.^2^ Although only a Type 1 pattern is diagnostic of BrS, there are recognized Type 2 and Type 3 patterns seen on ECG that may allude to the syndrome in the appropriate clinical context.

*SCN5A* is the gene most implicated in BrS. Autosomal dominant variants of the *SCN5A* gene that are ‘loss of function’ variants lead to a decrease in inward cellular sodium currents. This is manifested on ECG by the right precordial lead ST-segment elevation. Postulated aetiologies for the ECG changes observed with BrS pertain to the repolarization and depolarization hypotheses. The repolarization hypothesis is based on transmural dispersion of repolarization between the endocardial and epicardial aspects of the right ventricular outflow tract (RVOT).^4^ In contrast, the depolarization hypothesis explains the ECG findings in BrS through the relatively delayed electrical activation of the RVOT relative to the RV.^5^ Late and fractionated action potentials have been identified through electroanatomical mapping and appear to have a predominance in the epicardial RVOT in patients with a BrS.^6^ However, despite these hypotheses, pathogenic variants are found in only 20% of those who have the clinical phenotype seen with BrS.^1^ This low yield of genetic testing provides a challenge in diagnosing this syndrome. Therefore, a pharmacological challenge using a Singh–Vaughan Williams (VW) Class 1 antiarrhythmic is often used to provoke a Type 1 Brugada pattern on ECG which may otherwise only be seen transiently.

Exercise treadmill testing (ETT) is not currently performed as part of a standard diagnostic workup for BrS. This case series presents three unrelated patients who underwent ETT to evaluate what was later deemed to be non-cardiac chest pain. In these patients, a Type 1 Brugada pattern was evoked with ETT. Our observations suggest that valuable information can be obtained from an ETT, which can potentially serve as a diagnostic aid for BrS.

## Summary figure

**Figure:**
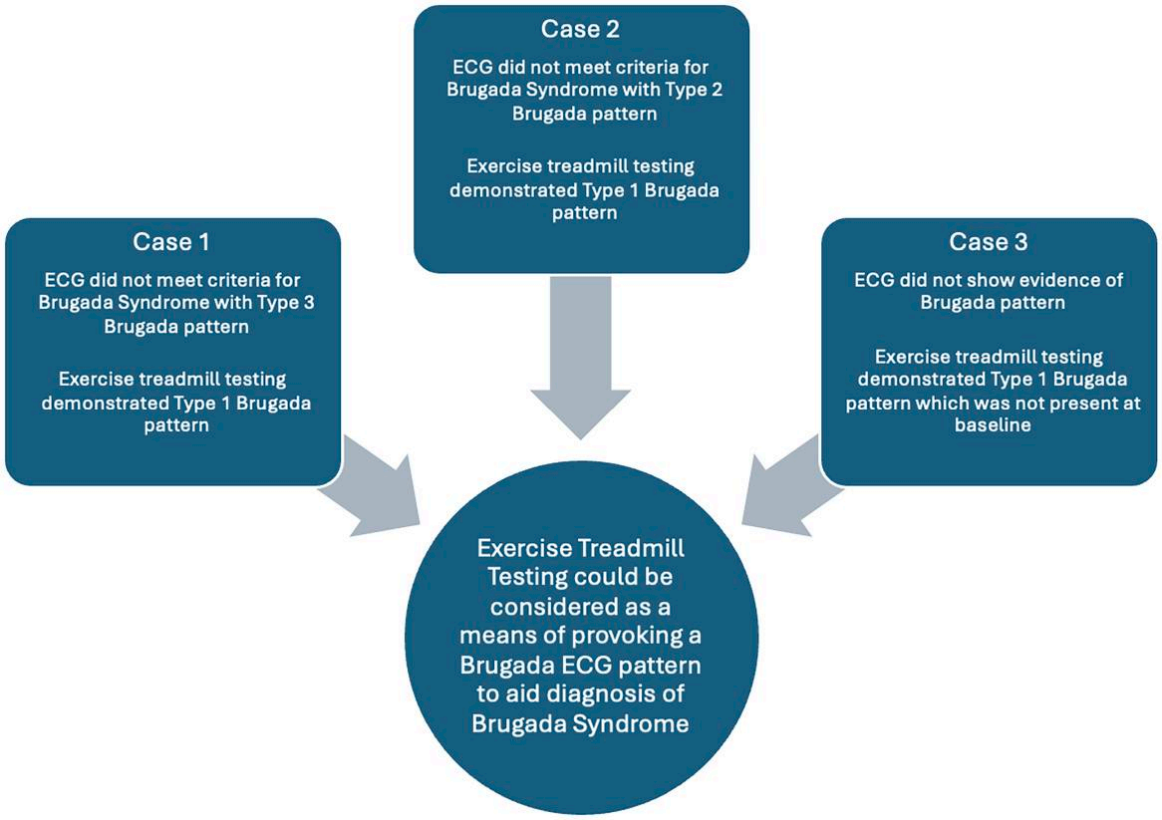


## Clinical case presentations

### Case 1

**Figure 1:**
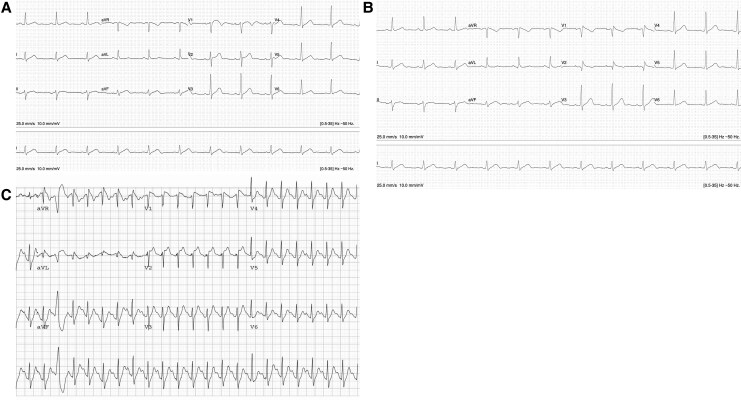
Case 1 Electrocardiograms. (*A*) Electrocardiogram with standard lead placement. (*B*) Electrocardiogram with high intercostal lead placement. (*C*) Electrocardiogram at 45 s into recovery phase on treadmill test demonstrating Type 1 Brugada pattern evoked with exercise. The patient exercised according to Bruce protocol^7^ for 10:14 min, achieving a work level of a maximum of 13.40 metabolic equivalents, with maximal heart rate 162 b.p.m. and maximal blood pressure 166/84 mmHg.

A 36-year-old Caucasian male was diagnosed with BrS during a clinical presentation for a febrile illness with a lower respiratory tract infection. Other than hypertension, he had no significant personal or family medical history of cardiac syncope, ventricular arrhythmias, or sudden cardiac death. His only medication was diltiazem ER 240 mg daily. Consistent with the known transient nature of the ECG changes associated with BrS, ECGs had not shown this Type 1 pattern. Electrocardiogram with standard lead position did not show changes observed with BrS, and high intercostal leads raised suspicion, but did not meet criteria for the diagnosis of BrS. As this patient had concurrently experienced atypical chest pain, he underwent an ETT according to standard Bruce protocol.^7^ This demonstrated a baseline non-diagnostic Type 3 Brugada pattern. He exercised for 10 min and 14 s, reaching Stage 4 on the Bruce protocol and achieving 13.4 metabolic equivalents (METs). His ECG transitioned into a Type 1 Brugada pattern that was most apparent early into recovery. There were no ventricular arrhythmias, nor were there any findings concerning for myocardial ischaemia (*Figure 1*).

Of note, he had no findings consistent with ischaemia or ventricular arrhythmia on ETT. Further workup included genetic testing with an 18-gene panel for BrS (Blueprint Genetics, Finland). This revealed he was heterozygous for a pathogenic variant in the *SCN5A* gene (p. Phe1617del; c.4850_4852delTCT), resulting in an in-frame deletion. This variant has been associated with BrS as well as long QT syndrome (LQTS): it has been established to cause both concomitant loss- and gain-of-function of the sodium channel.^8^ His response to ETT was not consistent with LQTS. As part of cascade screening, his two children received genetic testing and were found to be carriers of this variant. His 12-year-old son had a normal treadmill test, while his 14-year-old daughter has had deferral of her treadmill test due to a musculoskeletal injury. Neither child had a clinical phenotype for BrS.

### Case 2

**Figure 2:**
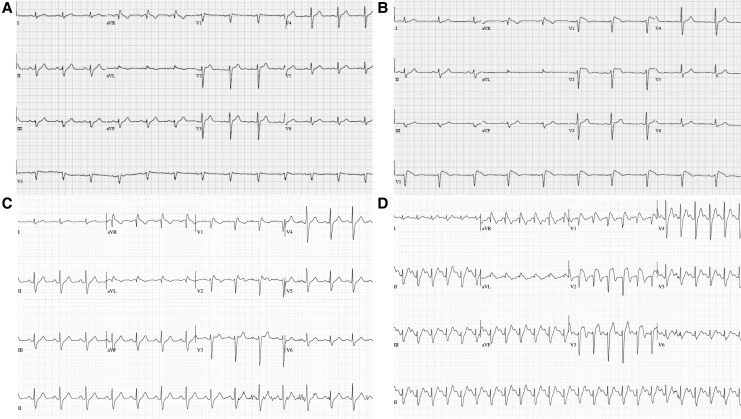
Case 2 electrocardiograms. (*A*) Electrocardiogram with standard lead placement 4 years prior to presentation. (*B*) Electrocardiogram with standard lead placement on presentation to the emergency department with atypical chest pain. (*C*) Standing electrocardiogram at rest prior to treadmill test commencement. *D*) Electrocardiogram at 50 s into recovery phase of treadmill test demonstrating a Type 1 Brugada pattern. The patient exercised according to Bruce protocol^7^ for 12:30 min, achieving a work level of a maximum of 15.20 metabolic equivalents, with maximal heart rate 164 b.p.m. and maximal blood pressure 176/100 mmHg.

We describe a 46-year-old Caucasian male who was incidentally diagnosed with BrS. This patient had no significant past medical or family history and was not on any regular or over-the-counter medication. ECGs with standard lead placement 4 years prior to presentation showed sinus rhythm without any evidence of Brugada pattern (*Figure 2*). He presented to the emergency department with atypical chest pain, and an ECG showed a Type 2 Brugada pattern at baseline. As part of his workup for chest pain, an ETT was performed. He exercised according to Bruce protocol^7^ for 12 min and 30 s, up to a maximum of 15.2 METs with no reproducible chest pain, evidence of ischaemia, nor sustained ventricular arrhythmia. Exercise treadmill test was stopped due to fatigue and dyspnoea. This patient developed a Type 1 Brugada pattern which was most apparent early into recovery and persisted through the recovery period for over 5 min (*Figure 2*). Occasional isolated premature ventricular contractions (PVCs) were seen on ECG in exercise and recovery. Genetic sequencing of SCN5A gene did not reveal any pathogenic variants.

### Case 3

**Figure 3:**
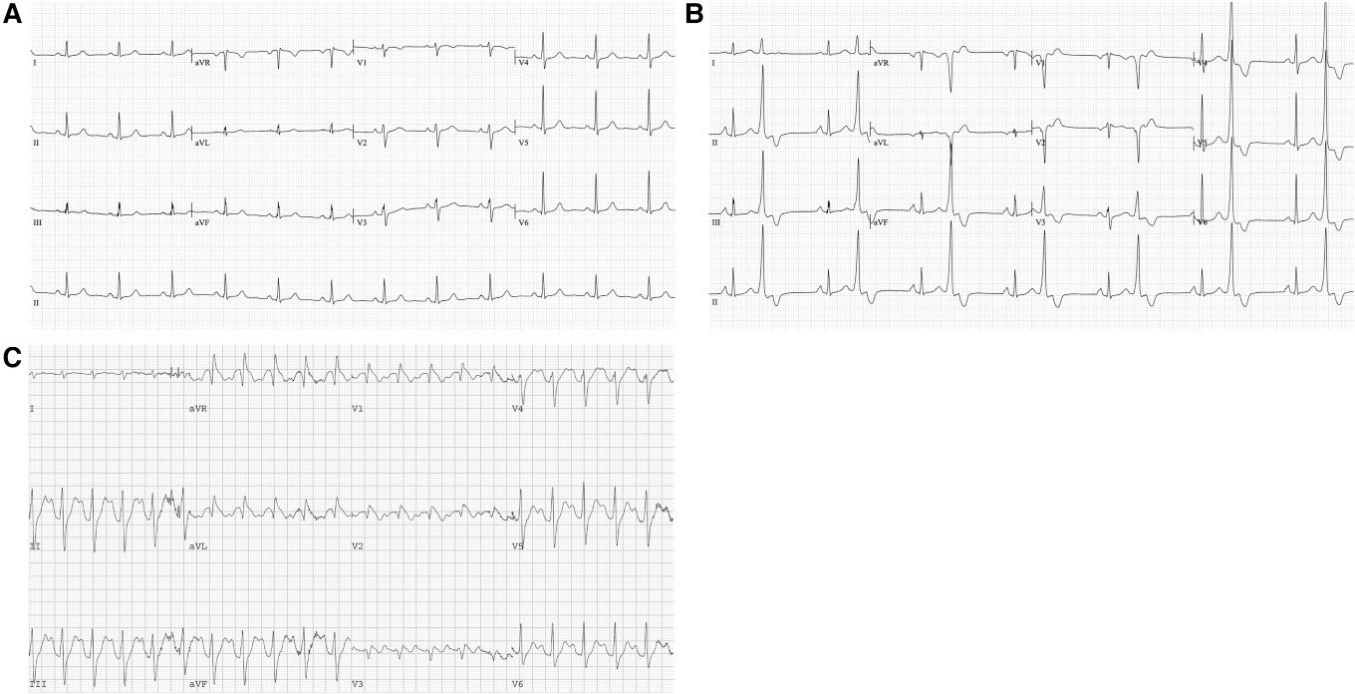
Case 3 electrocardiograms. (*A*) Electrocardiogram with standard lead placement. (*B*) Electrocardiogram with high intercostal lead placement. (*C*) Electrocardiogram at 50 s into recovery phase on treadmill test demonstrating Type 1 Brugada pattern. The patient exercised according to Bruce protocol^7^ for 04:11 min, achieving a work level of a maximum of 6.0 metabolic equivalents, with maximal heart rate 148 b.p.m. and maximal blood pressure 184/76 mmHg.

We describe a 66-year-old Caucasian female who had a high burden of monomorphic RVOT PVCs in an otherwise structurally normal heart with no significant coronary artery disease. This patient had been started on flecainide several years prior and had tolerated a dose of 100 mg twice daily with a good clinical response and reduction in PVC burden and symptoms. ECG with standard lead placement was unremarkable and high-lead placement did not show features concerning for BrS in conducted sinus beats. After a few years of starting flecainide, she developed atypical chest symptoms with activity, so a repeat ETT was performed. On ETT, she demonstrated a Type 1 Brugada pattern which was not present at baseline, most apparent early in the recovery period and persisting for 5 min into the recovery period (*Figure 3*). On reviewing her previous ETTs, this exact behaviour was observed 6 years prior yet overlooked. Upon this pattern being observed, we elected to cease her flecainide. Subsequent genetic testing was negative for a pathogenic variant in the *SCN5A* gene.

## Discussion

In this case series, we describe three patients where ETT was useful in demonstrating a Type 1 Brugada pattern most apparent in the early recovery phase immediately after peak exercise but not at baseline. Exercise treadmill test is not part of our standard workup for BrS, and these patients had an alternative indication for ETT. At baseline, they were not symptomatic with syncope, sudden cardiac death, or documented sustained ventricular arrhythmia, nor did they exhibit a family history of the same. Subsequent to ETT testing revealing Type 1 Brugada pattern and a diagnosis of BrS, these patients were managed conservatively with the avoidance of provocative medications such as sodium channel blockers, avoidance of binges of alcohol or food, and the early treatment of fevers.^9^

The hallmark protein recognized as the key causative component of BrS is the Na_V_1.5 cardiac sodium channel. This is responsible for the initiation and propagation of cardiac action potential through cardiac myocytes. Loss-of-function *SCN5A* gene variants, encoding the pore-forming α-subunit of the Na_V_1.5 cardiac sodium channel, explain approximately 20% of patients with a phenotypic pattern of BrS. As the presence of a loss-of-function *SCN5A* gene variant does not always result in a Brugada phenotype, a disease-causing variant alone is not diagnostic of BrS.^2^ Although there are other genes that have been associated with BrS, there is, at present, insufficient data to implicate these genes to be causative of BrS.^10^

Therefore, BrS remains a clinical diagnosis based on observed ECG patterns. Diagnosis is made challenging due to the transient nature of ECG changes. In order to provoke this pattern on ECG, a pharmacological challenge with a VW Class 1 antiarrhythmic medication, commonly flecainide, ajmaline, or procainamide, is performed. While these agents are all sodium channel blockers, this test is limited with results also dependent on the choice of agent. Out of the three commonly used agents, ajmaline is noted to yield the greatest number of positive results^11,12^ and is known to also act on potassium and calcium channels in addition to the sodium channel.^13^ Patients in the present case series had ECGs that did not exhibit a Brugada Type 1 pattern at baseline prior to ETT. In one of our patients, this had effectively occurred with the use of oral flecainide; however, the Type 1 pattern was only observed with treadmill testing.

ECG patterns reminiscent of BrS may be seen in various cardiac abnormalities including right bundle branch block, cardiomyopathy, and electrolyte disturbances.^14^ It is also important to differentiate BrS from Brugada phenocopies, which present identically on ECG to BrS, yet have other clinical causes.^15^ In our described cases, Brugada ECG patterns were detected in the absence of any electrolyte disturbances or Brugada phenocopies.

The mechanism to inform why such a Brugada pattern can be observed on an ETT is yet to be studied. It is possible that perhaps a rise in core body temperature during exercise or a use-dependent loss-of-function of the sodium channel may underlie these observations. Fever is known to induce a Brugada ECG pattern in patients with BrS, informing the management strategy of treating fevers early and aggressively. With ETT, it is with effort and perhaps a slight increase in core body temperature that we have observed the Brugada pattern on ECG. Vaughan Williams Class 1 antiarrhythmics are known to inhibit the sodium channel in a use-dependent manner, blocking the channel in its inactivated or open state. Conversely, the sodium channel blockade dissipates in diastole at a rate determined by the medication’s dissociation constant from the sodium channel. With tachycardia, there is a shorter diastolic interval and therefore increased sodium channel blockade at equilibrium. Thus, at higher heart rates, the degree of sodium channel blockade exerted by Class 1 antiarrhythmic agents is higher.^16,17^ Our case series demonstrates ETT evoking a Brugada Type 1 pattern on ECG in patients with and without Class 1 antiarrhythmic medication. Given the sodium channel encoded by the *SCN5A* gene is most implicated in BrS, it is conceivable that in certain cases, ETT may reveal the vulnerability of this channel with tachycardia, provoking a Brugada Type 1 pattern on ECG.

Therefore, this present case series is consistent with the concomitant literature in suggesting that ETT can be utilized to look for a Brugada pattern that may subsequently aid in the diagnosis of BrS. There are increasing reports of exercise unmasking a Brugada ECG pattern in asymptomatic patients.^8,18^ Moreover, a novel treadmill protocol using high precordial leads in the passive recovery phase of ETT has been recently proposed to improve diagnostic yield of BrS. A proposed mechanism to reveal the Brugada pattern was parasympathetic reactivation, thought to occur within the first minute of exercise cessation.^19^ Exercise treadmill test has also been described as a tool for prognostication of asymptomatic Type 1 BrS, using the duration of S wave upslope and degree of augmentation of J-point to suggest higher risk individuals.^20^

## Lead author biography


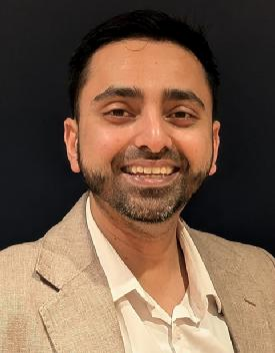


Ojas is a cardiologist in Melbourne, Australia. He had completed an electrophysiology subspecialty fellowship in British Columbia, Canada. He actively performs catheter ablation for atrial and ventricular arrhythmias. He has a keen interest in the pharmacology of antiarrhythmic medications and inherited arrhythmias.

## Data Availability

The data underlying this article are available from the corresponding author upon reasonable request.
